# Multiple Chronic Health Conditions and Their Link with Labour Force Participation and Economic Status

**DOI:** 10.1371/journal.pone.0079108

**Published:** 2013-11-01

**Authors:** Deborah J. Schofield, Emily J. Callander, Rupendra N. Shrestha, Megan E. Passey, Richard Percival, Simon J. Kelly

**Affiliations:** 1 NHMRC Clinical Trials Centre, University of Sydney, Sydney, New South Wales, Australia; 2 School of Public Health, University of Sydney, Sydney, New South Wales, Australia; 3 University Centre for Rural Health – North Coast, University of Sydney, Lismore, New South Wales, Australia; 4 National Centre for Social and Economic Modelling, University of Canberra, Canberra, Australian Capital Territory, Australia; Beijing Institute of Microbiology and Epidemiology, China

## Abstract

**Aims:**

To assess the labour force participation and quantify the economic status of older Australian workers with multiple health conditions.

**Background:**

Many older people suffer from multiple health conditions. While multiple morbidities have been highlighted as an important research topic, there has been limited research in this area to date, particularly on the economic status of those with multiple morbidities.

**Methods:**

Cross sectional analysis of Health&WealthMOD, a microsimulation model of Australians aged 45 to 64 years.

**Results:**

People with one chronic health condition had 0.59 times the odds of being employed compared to those with no condition (OR 0.59, 95% CI: 0.49, 0.71), and those with four or more conditions had 0.14 times the odds of being employed compared to those with no condition (OR 0.14, 95% CI: 0.11, 0.18). People with one condition received a weekly income 32% lower than those with no health condition, paid 49 % less tax, and received 37% more in government transfer payments; those with four or more conditions received a weekly income 94% lower, paid 97% less in tax and received over 2,000% more in government transfer payments per week than those with no condition.

**Conclusion:**

While having a chronic health condition is associated with lower labour force participation and poorer economic status, having multiple conditions compounds the affect – with these people being far less likely to be employed and having drastically lower incomes.

## Introduction

Around the globe, national populations are ageing. This is true of all OECD countries, and the trend is expected to continue into the future. In 2010, for example, between 13 and 17 per cent of the United States, United Kingdom and Australian populations were aged 65 years and over; by 2050 this proportion is expected to increase to between 21 and 26 per cent[1]. With an ageing population, the number of retired older individuals as a ratio of working-aged individuals is also increasing – this dependency ratio is currently at around 3 to 4 individuals of working age for every elderly retired person in the United States, United Kingdom, and Australia; however, by 2050 the ratio is expected to decline to less than 2 people in the labour force for every retired individual[1]. This makes it particularly important that individuals of working age actually participate in the labour force[2–4]. 

With the ageing of the population, older workers are making up an increasing proportion of the labour force aged population, making them a vital part of the workforce, however their labour force participation can be relatively low[5]. Ill health is a key reason for exiting the labour force early, particularly amongst older workers[6]. 

Numerous conditions, such as cardiovascular disease, diabetes, cancer and arthritis, which increase in prevalence with age [7], have been recognised for their potential to lead to withdrawal from the workforce [8–11]. However, many people with a long term health condition will suffer from multiple health conditions, especially amongst older age groups. It is recognised that those with multiple health conditions have poorer functional outcomes and a poorer quality of life [12–14]. While multiple morbidities has been highlighted as an important research topic[15], there has been limited research in this area to date, particularly on the economic status of those with multiple morbidities.

Persons with multiple chronic health conditions may be more likely to have their labour force participation affected than those with a single condition, or those with no health condition due to the additional functional restrictions faced. As such, persons with multiple chronic health conditions may be the most at risk of exiting the labour force early, particularly older workers who are more likely to suffer from multiple health conditions[12]. As a result of this lower labour force participation, individuals with multiple morbidities may also have lower incomes creating a large personal economic burden. This lower labour force participation may also result in significant costs to government from lower taxation revenue being received and greater outlays as government has to pay individuals higher support payments. 

This paper will assess the labour force participation of older Australian workers, aged 45 to 64 years, who have multiple chronic health conditions, single chronic health conditions, and no chronic health conditions to estimate the impact of multiple health conditions on labour force participation. It will then quantify the economic status – in terms of weekly private income received by individuals, the amount of weekly taxation paid by individuals, and the amount of government benefit payments paid to individuals – of people with multiple morbidities. While the results of this paper does focus on Australia, the results are generalizable to other developed countries as population ageing, low dependency ratios and low labour force participation of older workers are common issues facing government internationally[1–4]. 

## Methods

This study has been conducted using Health&WealthMOD, a microsimulation model of the 45 to 64 year old Australian population. The model contains detailed information, at the individual level, of health (including the type of conditions and number of conditions), labour force participation, and economic status. It has been successfully used in the past to document the economic impacts of various individual health conditions[16–23]. Health&WealthMOD was built upon individual record data from the 2003 Survey of Disability, Ageing and Carers (SDAC), a nationally representative survey of health and disability conducted by the Australian Bureau of Statistics[24], and STINMOD, a microsimulation model of income tax and government support payments[25,26], which is maintained and developed for the Australian Government by the National Centre for Social and Economic Modelling, University of Canberra. 

The base population of Health&WealthMOD was unit record data extracted from the SDAC, with the SDAC providing details on the number and types of health conditions suffered. From this dataset, individual records were extracted for those aged 45-64 years. The details extracted for each individual in the base population included demographic variables (for example, age, sex, family type, and state of residence), socioeconomic variables (level and field of education, income, benefits received), labour force variables (labour force participation, employment restrictions, retirement), and health and disability variables (main chronic conditions, number of chronic health conditions, general health status, type and extent of disability, support and care required).

Using STINMOD, additional economic information such as individual income, government support payments and tax liability was imputed onto the base data. This information was imputed onto the base population of Health&WealthMOD by identifying persons with similar characteristics on STINMOD and “donating” their income and wealth information onto Health&WealthMOD using a process, commonly used in microsimulation models, called synthetic matching[27]. Nine variables: sex (2 groups: males, females), income unit type (4 groups: single only, single with dependants, couple only, couple with dependants), type of government pension/support (3 groups: disability support pension, other pension/government support payments, no pension/government support payments), income quintile (5 groups: quintile 1, quintile 2, quintile 3, quintile 4, quintile 5), age group (4 groups: 45-49, 50-54, 55-59, 60-64), labour force status (4 groups: employed full time, employed part time, unemployed, not in the labour force), hours worked per week (5 groups: 1 - 15 hours, 16 - 24 hours, 25 to 34 hours, 35 - 40 hours, 41+ hours), highest educational qualification (2 groups: university, no university) and home ownership (2 groups: owner occupied, renting), that were common to both datasets and strongly related to income were chosen as matching variables for synthetic matching[28]. 

The data were then aged to reflect the 2009 Australian 45 to 64 year old population. The ageing was used to account for the disability and illness, demographic, labour force, earnings growth and other changes that had occurred between 2003 and 2009.

The variables used in this study regarding multiple morbidities came from the 2003 SDAC. The SDAC identified individuals as having no chronic health condition, a single chronic health condition only, and up to nine or more chronic health conditions. For ease of interpretation, this paper grouped those with four or more health conditions together. 

The 2003 SDAC recorded individual labour force participation. For those who stated they were ‘not in the labour force’, their main reason for not being in the labour force was recorded. Response options included: retired, study or returning to study, own ill health or disability, child care availability or children too young or prefers to look after them, too old, does not need or want to work, some else’s ill health or disability, other family considerations, pregnancy, lacks relevant schooling, training or experience, don’t know, and other. In this study those who were out of the labour force and stated their main reason for leaving the labour force was their own ill health or disability were considered to be ‘out of the labour force due to ill health’, from this it is also known that these individuals experienced poor health first and then left the labour force. Those who selected all other options were considered to be ‘out of the labour force due to other reasons’.

The variables used for private income, taxation payments, and transfer payments came from STINMOD. Private income consists of income from the following sources: wages/salary of main and second job; own business/partnership (pre tax but after expenses deducted); interest payments; dividends; property rent (net of expenses); royalties; other financial investments; superannuation and annuities; accident compensation and sickness insurance; regular workers' compensation; payments from persons not living in the household; child support and maintenance; overseas pensions and benefits.

### Statistical analysis

Initial descriptive analyses were carried out to estimate the number and proportion of individuals with no chronic health condition, one chronic health condition, two chronic health conditions, three chronic health conditions, and four or more chronic health conditions.

The labour force status of each of these groups was then assessed and logistic regression models were used to estimate the odds ratios and 95% confidence intervals (CIs) of being in the labour force (either full time or part time) associated with the number of chronic health conditions individuals have. Persons with no chronic health condition were used as the reference group and the results were adjusted for age, sex and the highest level of education. 

Descriptive analysis was then undertaken to determine the average weekly private income, taxation payments, and government support cash benefits attributable to individuals with no chronic health condition, one chronic health condition, two chronic health conditions, three chronic health conditions, and four or more chronic health conditions. 

A multiple linear regression model of the log of weekly private income was used to analyse the differences between weekly incomes of these groups. Analyses were repeated for weekly transfer income and weekly tax liability. The co-variates age group, sex and highest level of education were adjusted for in the regression models. Regression analyses were undertaken on log-transformed data in order to satisfy the assumptions of linear regression analysis, and the regression diagnostics confirmed that the assumptions were reasonably satisfied. 

The analyses were undertaken using SAS V9.1 (SAS Institute Inc., Cary, NC, USA). 

## Results

In Health&WealthMOD there were 8 864 individuals aged 45 to 64 years representing 5 423 900 individuals in the 2009 Australian population. Within this population, 58% had one or more long term health conditions. Table 1 shows the proportion of individuals with multiple long term health conditions for males and females in different age groups. There was a similar distribution of multiple health conditions across the age groups for males and females. The proportion of individuals with multiple long term health conditions increased with age for both males and females. 

**Table 1:** Multiple Long Term Health Conditions Amongst Males and Females of Different Age Groups - Australian Population Aged 45 to 64 Years, 2009.

	Number of Conditions	N	Number in population	% of population
*Females*				
45 - 54 years	0	1307	771 500	52%
	1	606	248 200	23%
	2	314	177 400	12%
	3	151	87 700	6%
	4+	220	107 900	7%
55 - 64 years	0	564	359 000	30%
	1	446	285 400	24%
	2	363	212 800	18%
	3	220	153 800	13%
	4+	314	190 600	16%
*Males*				
45 - 54 years	0	1316	767 900	51%
	1	586	344 700	23%
	2	289	183 400	12%
	3	168	100 500	7%
	4+	176	110 600	7%
55 - 64 years	0	593	391 500	32%
	1	460	310 500	25%
	2	299	214 100	18%
	3	216	138 700	11%
	4+	256	167 900	14%

Table 2 shows the proportion of individuals in the labour force and out of the labour force amongst those with varying numbers of long term health conditions. Over half of those with no chronic health conditions, one chronic health condition and two chronic health conditions were in the labour force. Only around one-third of those with four or more chronic health conditions were in the labour force – with the remainder out of the labour force because of their ill health or for other reasons. 

**Table 2:** Labour Force Status Amongst those with Multiple Chronic Health Conditions - Australian Population Aged 45 to 64 Years, 2009.

**Number of conditions**	**In the labour force**	**Not in the labour force due to ill health**	**Not in the labour force due to other reasons**
0	83%	0%	17%
1	71%	6%	23%
2	62%	10%	28%
3	49%	19%	32%
4+	34%	31%	35%

Of those who were not in the labour force due to ill health, 57% received a Disability Support Pension, 24% received another type of government pension or support payment, and 19% received no government support payment. Of those who were not in the labour force due to other reasons, 16% received a Disability Support Pension, 33% received another type of government pension or support payment, and 51% received no government support payment.

Table 3 shows the likelihood of being in the labour force for those with one or more chronic health conditions compared to those with no chronic health conditions, after adjusting for age, sex and highest level of education. Those with one, two, three or four or more chronic health conditions were all less likely to be in the labour force than those with no chronic health condition. Those who have one health condition had 0.59 the odds of being in the labour force than those with no health condition (OR 0.59, 95% CI: 0.49, 0.71). Those who have four or more chronic health conditions had 0.14 the odds of being in the labour force compared to those with no chronic condition (OR 0.14, 95% CI: 0.11, 0.18).

**Table 3:** Odd Ratio (OR) of Being in the Labour Force for those with Varying Numbers of Long Term Health Conditions, Adjusted for Age, Sex and Education - Australian Population Aged 45 to 64 Years, 2009.

**Number of conditions**	**OR of being in the labour force**	**95% CI**	**p-value**
0	REFERENCE		
1	0.59	0.49, 0.71	<.0001
2	0.43	0.35, 0.54	<.0001
3	0.29	0.22, 0.37	<.0001
4+	0.14	0.11, 0.18	<.0001

Table 4 is limited to people who were not in the labour force, for any reason. It shows for people with different health conditions the proportion with different numbers of co-morbidities. For example, the majority of people with diseases of the respiratory system who were out of the labour force had 4 or more chronic health conditions, and only 7% had no other co-conditions, while of those suffering heart disease or mental and behavioural disorders, 60% reported a total of 3 or more chronic health conditions. 

**Table 4:** Number of health conditions by condition type – limited to 45 to 64 year old population not in the labour force for any reason, 2009.

**Main health condition**	**Total number of chronic health conditions**			
	1	2	3	4+
High cholesterol	60%	14%	18%	8%
Asthma	35%	32%	16%	18%
Cancer	33%	10%	28%	28%
Arthritis and related disorders	29%	28%	17%	26%
Back problems	25%	18%	21%	36%
Diabetes	20%	20%	40%	20%
Depression	20%	17%	33%	30%
Mental and behavioural disorders	19%	21%	21%	39%
Heart diseases	14%	26%	16%	44%
Diseases of the respiratory system	7%	10%	21%	62%

Table 5 shows the value of weekly private income received, the amount of weekly taxes paid and the amount of weekly government benefit payments received by those with different numbers of chronic health conditions. The median value of weekly private income received declined with the increasing number of chronic health conditions an individual had. The median value of weekly private income for those with three chronic health conditions was less than half of that received by those with no health condition and less than half of those with only one health condition. The median value of private income received by people with four or more chronic health conditions was only $33 per week. The median value of tax paid by those with three or more health conditions was $0, and the median value of tax paid by those with two health conditions was one third of that paid by those with only one health condition. The median value of government benefit payments received by those with no health condition and up to three health conditions was $0, whereas the median value for those with four or more health conditions was around $250 per week. 

**Table 5:** Value of Weekly Private Income Received, Taxes Paid and Benefits Received by Individuals with Different Numbers of Chronic Health Conditions - Australian Population Aged 45 to 64 Years, 2009.

**Number of Conditions**	**Private Income**			**Taxes (inc. Medicare levy)**			**Benefits**		
	*Mean*	*SD*	*Median*	*Mean*	*SD*	*Median*	*Mean*	*SD*	*Median*
0	1,327	35,891	1,049	246	10,281	138	32	2,142	0
1	1,160	32,878	918	221	9,717	94	48	2,561	0
2	984	29,824	802	178	9,001	50	78	3,219	0
3	702	37,321	428	102	5,784	0	116	3,774	0
4+	440	21,245	33	58	4,389	0	181	3,571	248

*excluding those with negative incomes

Table 6 shows the age, sex and education adjusted difference in the value of weekly private income received by those with no chronic health condition and people with a single long term health condition and multiple health conditions, regardless of labour force participation. People with one chronic health condition received around one-third less in weekly private income than those with no health condition (-32.19, 95% CI: -45.10, -16.25). People with two chronic health conditions received less than half the amount in private income per week than those with no health condition (-52.77, 95% CI: -63.84, -38.32). People with four or more chronic health conditions received 94% less per week in private income than those with no health condition (-94.02, 95% CI: -95.71, -91.65).

**Table 6:** Difference in Weekly Private Income Received, Taxes Paid and Benefits Received Between Those with Different Numbers of Chronic Health Conditions, Adjusted for Age, Sex and Education - Australian Population Aged 45 to 64 Years, 2009.

**Number of Conditions**	**Private Income**					**Taxes (including Medicare levy)**					**Benefits**			
	*% difference*	*95% CI*		*p-value*		*% difference*	*95% CI*		*p-value*		*%difference*	*95% CI*		*p-value*
0	REFERENCE					REFERENCE					REFERENCE			
1	-32.19	-45.10	-16.25	0.0003		-49.32	-61.80	-32.74	<.0001		37.19	14.71	64.07	<.0001
2	-52.77	-63.84	-38.32	<.0001		-68.14	-77.80	-54.28	<.0001		168.0	107.81	245.63	<.0001
3	-80.81	-87.23	-71.15	<.0001		-90.13	-93.84	-84.20	<.0001		472.16	293.98	730.91	<.0001
4+	-94.02	-95.71	-91.65	<.0001		-97.47	-98.30	-96.22	<.0001		2,485.83	1,809.48	3,401.73	<.0001

Those with chronic health conditions paid significantly less in tax and received significantly more in weekly transfer payments than those with no chronic health conditions. Those with one chronic health condition paid around 50% less in tax per week than those with no health condition; similarly, those with four or more chronic health conditions paid nearly 100% less in weekly tax than those with no health condition. Those with one health condition received around one third more in weekly transfer income than those with no health condition, and people with four or more health conditions received around two thousand times more in weekly transfer income than those with no health condition.

## Discussion

More than half of the individuals in the older working age group (45 to 64 years) have at least one chronic health condition, and of those with at least one chronic health condition the majority suffer from multiple conditions. This highlights the importance of assessing the effect of multiple health conditions in this age group where ill health is a key reason for early retirement. This study has confirmed that those with multiple health conditions are far less likely to be in the labour force than those with no health conditions. Labour force participation decreases with increasing numbers of health conditions. 

In addition to the lower labour force participation, individuals with multiple chronic health conditions receive a weekly income of less than half of individuals without a chronic health condition and pay significantly less tax. People with multiple chronic health conditions also receive significantly more in government support benefits each week. As such, the lower labour force participation amongst those with multiple health conditions may translate into significant costs to both individuals and to governments.

Other studies that have looked at multiple health conditions have found that co-morbidities or multiple morbidities are associated with increased rates of disability[14,29] and poor physical functioning[30,31]. These findings have been supported by studies that have described the lower employment rates associated with specific disease combinations[32], and the loss of productivity associated with multiple morbidities[33]. However, these studies were not nationally representative. In contrast, our study is based upon nationally representative data and as such inferences about the entire 45 to 64 year old population can be made.

One of the main limitations of this paper is that it was conducted using cross-sectional data. While it is reported in the main data source, the SDAC, that those who retired due to ill health developed their health condition and then left the labour force, for those with chronic health conditions who left the labour force due to other reasons it is not known whether some members of this group may also have subsequently have developed a chronic health condition associated with their lower income that would have prevented their return to the labour force. Thus the results of this study may be somewhat conservative. However, the studies on this reverse relationship (the influence of income on health) have found the impact to be relatively small. Three studies utilising longitudinal data have found no significant relationship between income and self-reported health status [34–36], and others have found no significant difference for females [37–40]. Of the studies that did find a significant influence of income on health using longitudinal data, the magnitude of the change reported was very small. Jones and Wildman 2008 found that an increase of one unit in log income (equal to an increase of income of between £25,000 and £40,000) resulted in an increased probability of reporting ill health of 1% [41]; and Guasekara et al 2012 found that an increase of income by NZ$10,000 produces a 1% increase in the odds of reporting better health [42]. Numerous other studies also reported changes in health status of a similar magnitude [38,39,43–45].

The costs of retiring from the labour force prematurely due to ill health have been explored in numerous studies [46–51]. These studies assessed the impact of having a chronic health condition in general, but did not consider the compounding effect that multiple health conditions have. The current study has shown that the likelihood of labour force participation declines with increasing numbers of health conditions, and average weekly income also declines. Individuals with one health condition had weekly incomes 32% lower than those with no health condition, while- individuals with three or more chronic health conditions had approximately 80 % lower incomes than those with no health condition. As such, those with multiple health conditions should be the target of welfare strategies aimed at increasing the welfare of disadvantaged individuals. 

Within Australia, individuals who leave the labour force due to ill health may have access to a Disability Support Pension if they have medical confirmation that they cannot work due to a health condition and meet the eligibility criteria. The Employment and Support Allowance in the United Kingdom and the Supplemental Security Income scheme in the USA offer similar arrangements [52–54]. Despite this (limited) financial support from government, the financial burden of illness on individuals and their families will still be significant. It is documented that those who are dependent upon welfare as a primary source of income often have poor living standards and quality of life both within Australia [55,56]. 

Given the high proportion of people of older working age with multiple morbidities, it is important to understand the impact that multiple health conditions can have on labour force participation. In an ageing population, maintaining the workforce participation of individuals of working age is essential to maintain productivity and to support the rising costs of the growing number of older people [57–59]. As ill health is accepted as a key reason for early exit from the labour force, understanding the added complexities of multiple morbidities[15] is essential in any strategies aimed at preventing premature exit from the labour force. This study has demonstrated the lower labour force participation, the lower income, the lower taxation revenue and higher amounts of government benefits payments that are associated with individuals with multiple chronic health conditions.
